# Caregiver perceptions of usual care home programs for persons with acquired brain injury: a qualitative descriptive study

**DOI:** 10.3389/fresc.2024.1490874

**Published:** 2025-01-15

**Authors:** Elena V. Donoso Brown, Kasey Stepansky, Sarah E. Wallace, Isabella Bien, Emma Buttino

**Affiliations:** ^1^ Department of Occupational Therapy, Duquesne University, Pittsburgh, PA, United States; ^2^ Department of Communication Science and Disorders, University of Pittsburgh, Pittsburgh, PA, United States

**Keywords:** caregiver, acquired brain injury, home program, adherence, qualitative, usual care

## Abstract

**Objective:**

The study explores caregiver perceptions of home programs for clients with acquired brain injury based on current clinical care after transition to the community.

**Design:**

A qualitative descriptive study.

**Setting:**

Within the community, post inpatient rehabilitation.

**Participants:**

A convenience sample of eight caregivers of clients with acquired brain injury from one clinical site. All participants spoke English, were between the ages of 18 and 85 years, had no neurodegenerative disorders, and self-identified as caregivers.

**Procedures:**

Two nested semi-structured interviews were completed post-discharge from an inpatient rehabilitation facility. All interviews were audio recorded and transcribed. Qualitative data analysis was performed utilizing MAXQDA© software, consensus coding, and abstraction of themes.

**Results:**

Two themes with subsequent subthemes were identified: (1) Systems, Roles, and Responsibilities Influenced Caregivers' Perceptions of Home Program and Recovery Outlook and (2) Caregivers' Home Program Experience. The first theme addresses topics of caregiver roles and responsibilities, system supports and barriers, and their general outlook on recovery. Within the second theme, results provide a chronological description of home program training, use, and modification.

**Conclusions:**

A caregiver’s outlook on the care receiver’s recovery and home program implementation is influenced by the burden of responsibilities, and system-level supports and barriers. The home program experience of the caregivers was reported to involve limited but satisfactory training. Caregivers saw the value in home programs and advised others to engage in them. Future programs should encourage healthcare providers to provide explicit instruction to the caregiver about their intrinsic value to home program implementation and adherence.

## Introduction

As a leading cause of disability worldwide, an acquired brain injury (ABI) impacts the person who experiences the injury, and also the family around the individual, limiting engagement in meaningful activities and decreasing health-related quality of life for both the individual with ABI and the caregiver (1–4). To decrease this impact and maximize recovery potential, rehabilitation is typically recommended immediately post-injury (5, 6). However, persons with ABI continue to have limitations in returning to desired daily activities in part due to limited access to direct services. Investigation of the perspectives of clinicians and ABI survivors on service provision during post-discharge rehabilitation identified the need for improved quality of care in continuity of care, accessibility, information, and communication (7). It is common during usual care for rehabilitation therapists to extend services and support the continuation of rehabilitation through the assignment of home practice programs following discharge from inpatient rehabilitation (8–10).

Home practice programs in usual care are individualized therapeutic tools which outline exercises and activities to be completed unsupervised at home to support recovery (11–13). One factor that can substantially limit the potential impact of home programs is adherence to the prescribed program. Adherence has been reported as a challenge for persons with ABI as they often struggle to follow through with these home programs post-discharge from inpatient rehabilitation due to a variety of factors such as lack of equipment, decreased motivation, and fatigue (11).^1^

While the barriers persons post-ABI face are diverse, one factor that has been identified to support adherence to rehabilitation home programs is the perception of social support.^1^ In addition to being associated with self-reported adherence, occupational therapists and speech-language pathologists both identified the importance of another individual or support person as a factor that can influence home program adherence for persons post-stroke (9, 10). Although therapists and clients indicate that caregivers are important, only a few studies that were associated with randomized controlled trials explored the caregivers’ experiences with home programs as a part of recovery for persons post-ABI. Engaging in home programs as a dyad (i.e., caregiver and care receiver) has allowed the caregiver to experience increased preparation for discharge, and foster individualized tailoring of the home program (14). Furthermore, decreased caregiver burden was found when a dyadic home program for people with traumatic brain injury was tailored, individualized, and provided assigned responsibilities and carryover within the dyadic team (15).

While these sources emphasize caregiver involvement during home programs, our study seeks to explore caregivers’ perceptions of the value of home programs based on their personal experience and participation within them during usual care and outside of a controlled study environment. Additional information is warranted that represents if or how the experience changes over time as well as the experience across different care settings. This information can be leveraged by clinicians to inform their practice and by researchers looking to advance dyadic home-practice interventions. Therefore, the research question was how do caregivers of persons with acquired brain injury describe their experience with rehabilitation home programs provided during usual care?

## Methods

### Design

Utilizing a pragmatic paradigm, we intentionally selected a qualitative descriptive approach to obtain first-hand narratives and explore the experiences of caregivers in the training and implementation of home programs for clients with ABI (16, 17). This approach has been identified as an appropriate method when seeking perspectives on the experiences of services or programs. We choose this approach to provide an initial description of the caregivers’ experience with minimal interpretation given that the experience of usual care home programs has not been previously described. Standards for Reporting Qualitative Research were utilized to ensure transparency and appropriate synthesizing of caregiver reporting (18). This study was approved by the researchers’ institutions [IRB Protocol Duquesne University 2022/08/16, University of Pittsburgh 22010148]. Respondents gave written consent before starting interviews.

The research team consisted of two occupational therapists, one speech-language pathologist, and two occupational therapy students. All authors were centralized in an academic setting located in an urban area of Northeastern United States. All therapists had clinical experience working with persons with acquired brain injury and their caregivers. A shared assumption of the research team was that caregivers are critical to ABI recovery and that they frequently face challenges post-discharge from rehabilitation. In addition, we believed caregivers’ experiences and involvement with home programs would be highly variable.

### Participants

We utilized a criterion convenience sample to ensure that we sampled individuals who could best answer our research question. For inclusion in the study, participants had to personally identify as a caregiver for an individual with acquired brain injury who had received a rehabilitation home program and had to be between 18 and 85 years old. There were no requirements on the consent form defining the caregiver role, only self-identification. In addition, participants needed to speak English as their primary language. Participants were excluded if they self-reported a progressive neurological condition such as dementia or Parkinson's disease. We used two criteria to determine the sample size: (1) the number of participants we were able to recruit and (2) saturation. Saturation was determined during data analysis as no new codes or categories were identified.

### Procedures

Participants were recruited from a local rehabilitation hospital during or after their care recipient's stay. Potential participants were identified by therapists and only approached by the research team with the potential participant's permission. The rehabilitation hospital's standard practice included prescribing a home program before discharge; however, there were no consistent elements included within the home program and they were created based on the therapist's discretion. Before the interviews, participants completed an electronic consent form. Then, the initial interview was completed through a phone (*n* = 7) or video call (*n* = 1) between one to two months after discharge from the hospital. Two to three months later, participants completed the closing interview to identify any changes since the first interview. Changes reported by caregivers related to home programs, roles, and responsibilities occur over time (1, 3), thus it was important to consider both time points. All interviews were audio-recorded using computer technology via Zoom call (14 interviews) or QuickTime recorder (one interview).

### Materials

We used a research team-developed semi-structured interview guide to complete both sets of interviews. The topics were selected based on the researchers’ previous experience with the topic and knowledge of current literature on home programs and caregivers. The topics covered in the first interview guide included topics focused on home program training received, current participation in the home program, and outlook on the use of the home program. The second interview guide covered topics related to their role in the program at this new point in time, any changes related to the program, and revisited the topic of outlook on the home program. The guides were consistent during the study. Sample questions from the interview guides are in Table 1.

**Table 1:** Interview question examples.

Interview topics	Interview question examples (1. Initial/2. Follow-up)
Caregiver role	1. Can you tell me about your involvement in [care receiver's name]'s recovery process? 2. How would you describe your role now? Is it about the same or different?
Competence with home programs	1. How did [the hospital therapists] make sure you understood the exercises and activities? 2. If you could go back and ask any questions of the therapists [at the hospital] before you went home, what would you ask them?
Program-specific questions	1. Have you received any training related to that home program? 2. What are your thoughts on the home program that was given by [the hospital]?
Future outlooks	1. What are your thoughts on the value in home programs in general? 2. How do you think the rehabilitation home programs will work moving forward?

### Data analysis

Data were analyzed using inductive content analysis after all eight participants completed their interviews (19). Transcripts were imported into MAXQDA© for analysis. The team independently completed open coding of the first two participants’ initial interviews. The team then compared initial codes and generated an initial consensus codebook. A second comparison of codes occurred and any updates to the coding structure were made with consensus. This process of independent coding and team consensus was repeated for all the initial interviews. When the team moved to the second interview, the consensus codebook was again updated. Two research team members (EDB, KS) completed the abstraction of themes from the coded data. This process involved grouping similar codes into larger categories and then identifying the two main themes with corresponding subthemes.

#### Trustworthiness

Several methods were implemented to support the project trustworthiness (20). To ensure credibility and confirmability, before the analysis began each investigator engaged in a reflexivity activity to examine assumptions, beliefs, and judgments about caregivers and home programs. We also triangulated by analyst through individual coding followed by group consensus aimed to minimize the bias of any single analyst but also allow for a diversity of perspectives. Additionally, for confirmability, we completed member checking. A summary of the results was shared with four of the eight participants and they were asked for feedback. If something was reported to be missing, the analysis was checked to see if it was included in the larger picture. No revisions were made based on member checking. We have also provided information on the sample and the selection criteria used to allow others to consider the transferability of findings. Last, utilizing the MAXQDA© software allowed for an audit trail to be kept through the different file iterations as well as the merging of codes during abstraction supporting the study's dependability.

## Results

A total of 8 caregivers of clients with acquired brain injury were interviewed, including two males (25%) and six females (75%). All participants identified as white. Seven out of eight participants (88%) participated in both interviews. Seven participants were spouses of an individual with ABI. The average interview length was 20 min. Additional participant demographics including education, work status, and caregivers’ reported impairments of their care receiver resulting from the ABI are in Table 2.

**Table 2:** Participant demographics (*n* = 8).

Pseudonyms	Gender	Age	Education level	Work status	Nested interview completion	Relationship to care recipient	Caregiver perceived impairments of care recipient during interviews
Thomas	Male	NR	High School	Retired	2 of 2	Spouse	Physical, cognitive, fatigue
Cynthia	Female	63	1 Year of College	Retired	1 of 2	Sister	Physical
Jeff	Male	70	Bachelors Degree	Retired	2 of 2	Spouse	Physical, cognitive, endurance
Amy	Female	NR	2 Years of College	Part-Time	2 of 2	Spouse	Cognitive
Deb	Female	72	Masters Degree	Part-Time	2 of 2	Spouse	Communication
Kim	Female	67	Doctoral Degree	Retired	2 of 2	Spouse	Physical, mental health
Sue	Female	62	Masters Degree	Retired	2 of 2	Spouse	Physical, cognitive
Carol	Female	70	High School	Retired	2 of 2	Spouse	Physical, cognitive, communication

NR indicates age not reported, but participant reported between ages of 18–85 years.

The two key themes that captured the data were: (a) *Roles, Responsibilities, and Systems Influenced Caregivers’ Perceptions of Recovery Outlook* and (b) *Caregivers’ Home Program Experience*.

### Roles, responsibilities, and systems influenced caregivers’ perceptions of recovery outlook

The first theme reflected the factors that caregivers reported impacting their lives after discharge from inpatient rehabilitation that were not directly connected to home programs but that influenced their experience during their care receivers’ recovery. These factors included the following subthemes (1) caregiver roles and responsibilities, (2) system supports and barriers, and (3) perceived impairments and recovery outlook.

#### Caregiver roles and responsibilities

Multiple participants indicated that their roles and responsibilities as a caregiver increased upon discharge home to maintain care, home maintenance, and finances. Carol said, “There's just so much more I do for him now that he used to do himself…around the house or whatever”. Kim similarly reported, “He wouldn't be able to be home if I wasn't here.” Deb noted finance needs, “It was thrust upon me to figure out all the finances.” Thomas spoke to the sudden nature of this responsibility, “This was like a curveball that came out of nowhere.” Collectively these comments illustrate how caregivers experienced increased responsibilities that, at times, felt overwhelming after a family member's brain injury. Caregiver perceptions were also influenced by the caregiver and care receiver's previous roles prior to the injury, the caregiver's availability, and previous experience with health care. Seven of the eight caregivers spoke to the significant role change because their care receiver was independent with all daily activities and/or working before the recently acquired brain injury. Only Jeff stated that his role did not change too much because his care receiver had medical concerns prior to the recent ABI.

#### System supports and barriers

Caregivers described multiple ways that they were supported by the current or previous medical team. Kim stated, “The in-home [therapists] were all extremely helpful…sometimes saying okay so you’re having trouble getting out of bed lets walk back to bed or find a better way to do this.” Deb stated, “I'm very grateful that we have… this physical therapist as well as speech coming here to you know give these tips and pointers and make sure he's doing what he's supposed to be doing”. Caregivers also indicated systemic barriers compound the burden of new roles and responsibilities they were navigating. Thomas stated, “I tried to get more [therapy] in home, but the system doesn't want to pay for the services.” Jeff stated, “I think if they would have looked at her whole situation and there were decisions that were made that could have been made differently that would have made her safer in the environment that she was going into.” Deb also revealed difficulties with system navigation, “I never knew about private insurance. And then on top of the bills, it's like the short-term disability, the long-term disability, and the stuff insurances need.”

#### Perceived impairment and outlook on recovery

In addition to more responsibilities, prior experiences, systematic supports, and barriers caregivers reported on their perception of their care receiver's impairments and overall recovery. Five out of the eight participants noted impairments in more than one area that remained post-discharge from the hospital (See Table 2). During interview one, three participants expressed mixed opinions of recovery often with recognition that there was room for improvement. Deb stated, “He's come such a long way. but he has a ways to go.” Two participants expressed negative perceptions of recovery. For example, Carol stated, “To be honest, I'm not seeing any big improvement”. However, Kim was optimistic about recovery during the first interview stating, “He's been good and it's getting better each day.” During the second interview, the outlook had improved for most of the participants (*n* = 5) reporting improvement. Sue stated, “I was pleasantly surprised at how his cognition has now come back, not 100% but 85%.” However, some participants continued to have a mixed or negative outlook during the second interview Carol stated, “There hasn't been much improvement”. Similarly, Jeff noted in the second interview, “I'm going to have to take her to the doctors because she's having some like memory lapses … I think she really needs to be reevaluated in some capacity.”

### Caregivers’ home program experience

The second theme focused on the description of the home program experience from the caregiver's perspective. This included subthemes of (1) caregiver home program training, (2) content and environment for home program use, (3) caregiver support of home programs, (4) facilitators and barriers, (5) future home program use, and (6) value and advice.

#### Caregiver home program training

When describing their experience with home programs, caregivers were asked to begin by describing their experience with training on the home programs. Caregivers could often describe elements of training that occurred at the hospital that were non-specific to home programs. For example, Sue noted, “Yeah they also showed me you know like how he should get up and down from the bed, off the bed, into the car, how he should do that.” When redirected to discuss the home programs, many caregivers reported limited training. Cynthia reported that the amount of training was not adequate to ensure understanding. Amy noted, “I didn't really get much training at [hospital] it was kind of, I don't know, seemed a bit hurried when he was getting out of there.” It was also reported that what occurred was not perceived as training. Deb states “Um, I didn't really, she didn't really train me. I would not call it training.” Despite the limited nature of the training provided, participants reported satisfaction with the training. Thomas said, “Yeah, it was one day, but that was quite sufficient.” Some participants noted elements like handouts were supportive. See Table 3 for more reported training from each participant.

**Table 3:** Home program training, content, and reported follow-up (*n* = 8).

Pseudonyms	Caregiver reported home program training	Home program training satisfaction	Caregiver description of home program elements	Therapy home visits	Communication with therapy team
Thomas	Yes	“Adequate”	Leg exercises and puzzles	Yes	Requested more time for both in-home and outpatient therapies
Cynthia	No	Did not report	Leg exercises and walking	Did not report	Did not report
Jeff	No	“The way they explained the exercises was really good”	Band exercises	Yes	Similar expectations as inpatient therapists—Advocated for a reevaluation.
Amy	No	“Well prepared”	Physical therapy exercises, puzzles and brain teaser workbooks	Yes	Individualized to return to work activities
Deb	No	“I have no problems with it”	Walking, resistance training, writing	Yes	In-person and on ZOOM calls
Kim	Yes	“Quite sufficient”	Walking, workbook for cognition, general exercises, arm stretches	Yes	Advocated for more individualized care
Sue	Yes	“We felt comfortable with what we needed to do”	Walking, resistance bands, balance exercises, stretching, hand putty exercises	Yes	Use of an app for home program guidance
Carol	No	“Just enough”	Communication workbook	Yes	1–2 times a week

#### Content and environment for home program use

When asked about the home program's content, some participants noted that there was not much to them or their difficulty level was low and repetitive. For example, Carol reported, “He really wasn't given much of anything when he left there.” Cynthia noted her care receiver had been given “.. leg lifts and stuff like that, like moves, you know, side to side.. walking.” Caregivers in this sample did not report any difficulties with finding space, equipment, or working the home program into the daily routine. All of the caregivers indicated that they felt the home program had a feasible number of exercises and enough time to complete them. Some did qualify saying that they had enough time because they were retired. Most caregivers reported that equipment was provided to them by the healthcare system, however, one did mention that they had to personally pay for home equipment.

#### Caregiver support of home programs

The most frequently reported method of support for home programs was through verbal encouragement. Verbal encouragement included caregivers providing reminders to complete the home programs and encouragement to complete the program. Cynthia stated “Oh, I always encouraged him. Tell him that you know it is for his own benefit.” Others reported providing more direct setup or supervision. Amy reported, “..I liked to make sure he sits for two hours a day and works in that book.” A few participants reported needing to be there to physically support the home program recommendations. Carol reported, “But now he needs me more and more because when he gets up to walk, I sort of have to go with him.” Three participants acknowledged a change in their role in home program support between the two interview time points. See Table 4 for examples.

**Table 4:** Thematic summaries and quotations.

Theme 1: Systems, roles, and responsibilities influenced caregivers' perceptions of home program and recovery outlook		
Subtheme	Summary of codes	Representative quotations
Caregiver roles and responsibilities	Increased care responsibilities related to their care receivers’ recovery	Deb: “It was overwhelming”
		Thomas: “It's a change of lifestyle.”
		Jeff: “The transition from husband and wife to patient and caregiver…happens gradually, in a sense then you get to the point where you realize that that's the way it is.”
	Previous experience, roles, and availability	Kim: “I would not be able to do this if I wasn't retired.”
		Amy: “By the time I got down there after work, all the therapists were gone.”
		Deb: “I was in healthcare before so I kind of knew the drills”
System supports & barriers	Supports: rehabilitation staff and facilities	Thomas: “Because when she's up there in the facility… they’re pushing her more than what she would be doing the exercises at home.”
		Carol: “And since he's been home, he has an excellent speech therapist. She's just so good. You know, we’re really pleased with her”
	Barriers: systematic navigation, payment, additional resources	Jeff: “The only negative part that I would say, like I said earlier is the follow up… It would be nice just to have someone say, “Oh how are you doing?”
		Carol: “They just changed his speech therapist from two days to one, which I’m not thrilled with, but she said it has to do with insurance and everything.”
		Deb: “My gut feeling is that it is not going to last long enough…whatever they approved him for six months is about it. It could be some improvement after that, but that's when short term disability stops.”
		Thomas: “We went back to the doctor, I just wanted more [rehab services].”
Perceived impairments & outlook on recovery^a^	Uncertainty in prognosis and recovery timeline: concerns about recovery	Carol: “I’m getting to the point where I don't know if it's going to come to him.”
		Thomas: “Her reflex or response time is still not good enough for her to drive a vehicle. I just hope it all works. Hope it gets better.”
	Positive trajectory with recovery	Amy: “He's getting better, but it's a slow process.”
		Kim: “His level of independence in home continues to increase”
		Deb: “His signature came along really well. I was proud of that, because he does have a lot of papers to sign.”

Theme 2: Caregivers’ home program experience		
Subtheme	Summary of codes	Representative quotations
Caregiver home Program training	Elements of training	Thomas: “They showed me how to handle her on the steps and stairs and in and out of the shower”.
		Sue: “One day the PT director showed me um when he was using the cube with the numbers on it.”
	Limitations in training	Deb: “I was given explanation about why they were doing whatever therapy he was doing at the time.”
		Jeff: “I really don't remember them showing the any kind of training I basically, just talked to her about what they were doing with her and stuff.”
		Amy: “I mean, they gave us a lot of literature, but as far as like, you know, talking to me, or anything no, I didn't get that.”
	Adequate or satisfactory	Sue: “I think it was just enough for me anyway.”
		Kim: “We came home with written things and that helped because I needed to remember everything he was supposed to do.”
		Amy: “I was comfortable with, you know, what we're doing and all of that.”
Content of and environment for home program use	Low difficulty	Kim: “To be honest he didn't seem to find any of them difficult you know…The things with his balance, he used resistance bands too as he went on.”
		Thomas: “Standing behind a couch or raising your leg of things like that.”
	Repetitive	Deb: “He had a book that he would keep it in so he could you know, write it over and over again. Look at it, write it over and over again. And that was part of it, his exercises for speech.”
		Carol: ”She has made up parts of different words and he's just to say those over and over again.”
	Supportive context	Sue: “Since it's a ranch home, our living room/dining room area is really nice and level and flat and you can look out and see the woods.”
		Kim: “There's a railing that works well for the ones where you stand and hold on…he knows which spaces are suitable.”
Caregiver support of home programs	Verbal encouragement	Thomas: “Yeah, I’ll ask her if she's done them.”
		Deb: “…he needs more reminders than anything.”
	Supervision	Carol: “I just tell him slow down and take your time.”
		Kim: “I don't feel like I need to be watching, except for this one set that he cheats on. (laughs). So, I need to be there to make sure he slows down.”
		Sue: “I see myself as like the guide and the you know supervisor of his program.”
	Physical support	Jeff: “There's a couple exercises she does have to have the second person with and I help her with that.”
		Kim: “I get him on his exercise machine. My job is there to help him get on it and help him get off it.”
	Change over time^a^	Sue: “… so it went from I had to participate, and you know kind of get him into it and fired up. And then my role now is … just reminder.”
		Kim: “I’m doing less and less.”
		Jeff: “It really hasn't [changed]. I would say it's gotten a little bit more intense.”
Home program facilitators and barriers	Facilitator: independent motivation	Thomas: “The person's gotta want it; have that drive to get better… it's the mentality as a person that's going to affect it.”
		Carol: “He has trouble motivating himself”
		Kim: “It would be a whole lot harder if he wasn't motivated.”
		Jeff: “If the person that's receiving [the home program] isn't receptive and doing what they want them to do they’re not going to make any progress. It's up to the individual.”
	Barrier: emotional reactions	Thomas: “I think it's a little bit more boring at home” and “What really concerns me is the mental side of things. Sometimes she gets aggravated.”
		Sue:” I don't know if he forgets or if he just doesn't want to do those other ones at home.”
	Barrier: physiological concerns	Carol: “He started to get all of these dizzy spells”.
		Deb: “He needs to learn he can't, you need to challenge yourself, but you need to rest too. But sometimes he's pushing himself too hard.”
		Sue: “I think with his current state of mind that he forgets about that he has to do the program exercises.”
Future home program use^a^	End of first interview	Thomas: “I think it's going pretty good, considering it's only been two weeks since the incident.”
		Kim: “I’m sure he's going to keep doing them.”
	End of second interview	Sue: “I think he’ll do them…I think that he’ll only do certain sets, probably his favorite ones.”
		Jeff: “Well, she's been doing the exercises. Not as readily as she had been doing them, but she's still doing them.”
	Shift to meaningful activity and uncertainty	Amy: “He had difficulty with numbers. So I said, do poker and blackjack that's counting numbers.”
		Thomas: “We go somewhere every day, get her out of the house. Um, I'd say, trying to keep her busy with this part time job she had, where she's a real estate agent.” and “Last Wednesday night, I took her to bingo.”
		Jeff: “I think they inferred she was supposed to do them forever and ever and ever. But they didn't come out and say, you know you really need to keep these going.”
Home program value and advice	Benefit and value	Amy: “I think it's very important”
		Deb: “Um, very valuable yeah.”
		Thomas: “I think, I think that the uh that the program was good, but its better when there's somebody there to push patients to work to work harder at it.”
	Advice to other caregivers	Deb: “I would say, don't delay on anything, start things immediately. Just keep working on them.”
		Carol: “Just try to help them as much as possible. Be there for them…You know, encourage him all the time. And tell him you know, don't worry. And you know, it’ll come to you.”
		Amy: “I’d just tell them to keep at it. It works. It, it may take a while but it works.”
		Jeff: “I would just say, try and make them be as motivated as possible to do them.”

^a^ Denoted subthemes with changes between first and second interview.

#### Facilitators and barriers

In addition to the way they participated in the home program, caregivers also offered insight into the perceived facilitators and barriers around home program engagement by their care receivers. Caregivers perceived the independent motivation of the care receiver as a primary facilitator of home program success. Participants Amy, Deb, Kim, and Sue all indicated that they thought their care receiver was motivated and that helped with the follow-up of their home programs (Table 4). Perceived home program barriers reported by caregivers were diverse. For example, some participants noted emotional reactions of frustration or boredom. Carol stated, “He just gives up because he's so frustrated.” Other caregivers mentioned physiological barriers limiting home program engagement. Jeff said, “She really doesn't have as much stamina as she used to.”

#### Future home program use

Caregivers were also asked about their outlook on the home programs moving forward at the end of each interview. At the end of the first interview, participants reported feeling capable of completing the home program and they would continue using it. During the second interview, more participants reported a shift in what was being done around home programs. For example, Carol reported a focus on activities from a specific discipline, “It's basically speech..like grade schoolbooks to help his phonics.. it's good practice for him.” Others reported a shift to engaging in activities in their daily lives more than the prescribed home program. Kim noted, “it's become a more natural part of life rather than let's stop and exercise.” See Table 4 for additional examples from the first and second interviews.

#### Value and advice

Last, caregivers reported on the value of home programs and offered specific advice to other caregivers about home programs. All participants found value in the use of the home programs. Kim reported “[Home programs] were really important even mentally for him to just have something to do.” When asked to provide advice to others who might be a caregiver for someone after a stroke and the use of home programs, participants focused on working to engage the clients in the programs immediately upon return home and providing encouragement. Sue said, “Do it the minute you get home, don't, don't let, don't let them get complacent by not doing them.” Other pieces of advice focused on the use of novel treatments like a hyperbaric chamber (Deb), the importance of a power of attorney (Deb), and supervision and safety (Thomas).

## Discussion

This qualitative study describes the perceptions of caregivers whose care receiver post-ABI was prescribed a home program as part of usual care before being discharged from inpatient rehabilitation. A caregiver's outlook on the person with ABI's recovery and home program implementation appears to be influenced by the presence of additional responsibilities, availability of the caregiver, and system-level supports and barriers. In addition, the caregiver's home program experience involved limited but satisfactory training, and home programs that were repetitive and easily done at home. Caregivers offered encouragement and occasional assistance if needed by their care receiver. Caregivers saw the value in home programs and advised other caregivers to support persons post-ABI in completing home programs.

The additional roles and responsibilities reported by caregivers often led to a need for additional support. Caregivers identified a desire for additional therapies, follow-up to care, and support navigating the healthcare system. The availability of the caregiver to support the client with ABI is also critical for successful discharge home and continued rehabilitation and recovery (21). Limitations in familial caregiver support can negatively impact long-term health outcomes for clients post-ABI. Several participants reported challenges navigating the system and a need for additional support which highlights the previously established need for the development of a standard care pathway to better support caregivers (22). Current usual care would benefit from continued development and study of evidence-based navigation support programs post discharge that are clear and easy to use for clients with ABI and their families (22). Providing additional support to navigate systematic barriers may allow caregivers to be more involved with home program implementation.

The second theme described the experience of home programs from the caregivers’ perspective and while caregivers endorsed the value of home programs, they often described them as separate from their other caregiver roles and responsibilities. Caregivers typically identified their role related to home programs as being an encourager of the person to participate rather than actively engaged in the program itself. Despite evidence supporting caregiver involvement increasing adherence (23, 24)^1^, often caregivers in this study instead focused on the person with ABI's individual motivation levels or need for additional rehabilitation professional support. While the individual motivation of the person with ABI has been identified by practitioners as a key moderator for home program implementation (9), individual motivation has the potential to be enhanced by the additional supports that a caregiver can provide during home programs (25). Therefore, therapists then should consider the use of dyad interventions as a means to support caregiver participation in home programs. Dyad interventions (26) actualize the engagement of both the caregiver and care receiver and could increase participation in home programs to support the health and well-being of both individuals. The active involvement of the caregiver in the home program plan is important as dyad intervention studies that intentionally engage the caregiver and care receiver in a shared goal or activity have demonstrated better outcomes than teaching caregivers to deliver home program exercises.^2^

Limitations of this study include only recruiting caregivers and sampling from a single clinical site for recruitment. Additionally, the experiences of the caregivers sampled within this study may not be reflective of other informal caregivers from different racial and socioeconomic backgrounds. All caregivers were either employed part-time or retired, therefore, this study does not reflect the experiences of informal familial caregivers who are working full-time or working in multiple role capacities, which may limit transferability. Future studies should diversify clinical settings for recruitment, as well as sample for the experiences of other familial caregivers such as children, friends, neighbors, or more distant relatives. Our study only recruited one caregiver from a non-spousal relationship so relationship diversity will be an important component of recruitment for future studies (27).

Several considerations and future directions for clinical practice and for research can be drawn from these results. For clinical practice, quality improvement initiatives are needed to help translate evidence of integrated and holistic dyad-focused education on home programs and the subsequent impact on adherence. Supporting caregivers as they navigate their novel roles and responsibilities should be a part of the caregiver training process, starting during hospital rehabilitation (22). Explicit instruction as a part of the home program training to the person's social support network about their intrinsic value to home program implementation is critical to support adherence.^1^ These instructions should take into account the caregiver's roles, relationship with care receiver, availability, and additional responsibilities. Our study focused exclusively on the caregiver experience during usual care, however future investigations could explore the dyadic experience. In addition to expanding the understanding of the home program experience, this would also allow for more detailed information to be gathered from the medical record of the person with an ABI.

## Conclusion

In conclusion, our study describes that the additional caregiver responsibilities and system-level supports and barriers may influence a caregiver's outlook on the care receiver's recovery. In addition, caregivers saw the value in home programs despite limited training and advised other caregivers to ensure engagement in home programs. Future initiatives should give pathways to healthcare providers to ensure explicit instruction to the caregiver about their intrinsic value to home program adherence.

## Data Availability

The raw data supporting the conclusions of this article will be made available by the authors, without undue reservation.

## Footnotes

^1^Donoso Brown EV, Wallace SE, Tichenor SE, Foundas B, Blemler R. Determining predictors of self-reported adherence to rehabilitation home programs for persons with acquired brain injury: a prospective observational study. NeuroRehabilitation.

^2^Kringle EA, Kersey J, Kim GJ, Bhattacharjya S, Dionne TP, Farag M, et al. Dyad interventions for health-related quality of life, activity, and participation after stroke: a systematice review. Disabil Rehabil.
